# Multimodal abnormalities of brain function in chronic low back pain: a systematic review and meta-analysis of neuroimaging studies

**DOI:** 10.3389/fnins.2025.1535288

**Published:** 2025-02-05

**Authors:** Xingyao Chen, Nuo Chen, Peng Lai, Yiqi Sun, Jie Yu, Ming Xin, Deliang Zhu, Fanrong Liang, Qian Song, Shirui Cheng, Zhengjie Li

**Affiliations:** ^1^The Acupuncture and Tuina School, Chengdu University of Traditional Chinese Medicine, Chengdu, China; ^2^National Clinical Research Center for Chinese Medicine Acupuncture and Moxibustion, First Teaching Hospital of Tianjin University of Traditional Chinese Medicine, Tianjin, China; ^3^Acupuncture & Brain Research Center, Chengdu University of Traditional Chinese Medicine, Chengdu, China; ^4^Department of Traditional Chinese Medicine, Chengdu Fifth People’s Hospital (The Second Clinical Medical College, Affiliated Fifth People’s Hospital of Chengdu University of Traditional Chinese Medicine), Geriatric Diseases Institute of Chengdu/Cancer Prevention and Treatment Institute of Chengdu, Chengdu, China; ^5^Department of Rehabilitation, Chengdu Fifth People’s Hospital (The Second Clinical Medical College, Affiliated Fifth People’s Hospital of Chengdu University of Traditional Chinese Medicine), Geriatric Diseases Institute of Chengdu/Cancer Prevention and Treatment Institute of Chengdu, Chengdu, China; ^6^Key Laboratory of Acupuncture for Senile Disease (Chengdu University of TCM), Ministry of Education, Chengdu, China

**Keywords:** chronic low back pain, functional magnetic resonance imaging, spontaneous brain activity, meta-analysis, resting-state

## Abstract

**Objectives:**

Neuroimaging investigations into chronic low back pain (CLBP) have detected functional abnormalities across a spectrum of brain regions, yet the findings have often been inconsistent. In this meta-analysis, we integrated the existing data, delineating a pattern of coherent results from the encompassed studies.

**Methods:**

A systematic search of neuroimaging studies investigating the brain activity differences between CLBP and Healthy controls (HCs) was conducted in seven databases up to December 22, 2024. An anisotropic effect-size signed differential mapping (AES-SDM)-based meta-analysis was carried out to report the results and perform a multimodal analysis.

**Results:**

A total of 20 publications reporting on 24 experiments in this meta-analysis. The ReHo meta-analysis showed abnormal spontaneous activity of left inferior temporal gyrus (ITG), left superior frontal gyrus (SFG), right middle frontal gyrus (MFG), right precuneus, right fusiform gyrus and bilateral postcentral gyrus (PoCG) in CLBP patients. The ALFF meta-analysis demonstrated functional alterations in the right rolandic operculum (extending to the right insula and right IFG), left ITG, left middle occipital gyrus (MOG), left paracentral lobule, left PoCG and bilateral cuneus cortex in CLBP patients. The results of the functional group meta-analysis revealed that patients with CLBP displayed new decreased functional activity in the right thalamus, right precentral gyrus (PreCG) and right lingual gyrus.

**Conclusion:**

Patients with CLBP exhibit extensive multimodal functional neuroimaging abnormalities, involving brain regions related to pain perception, emotional processing, cognitive functions, and both the visual and motor cortices. These meta-analysis findings might provide a valuable reference for the identification of potential therapeutic targets for CLBP in the brain.

## Graphical abstract

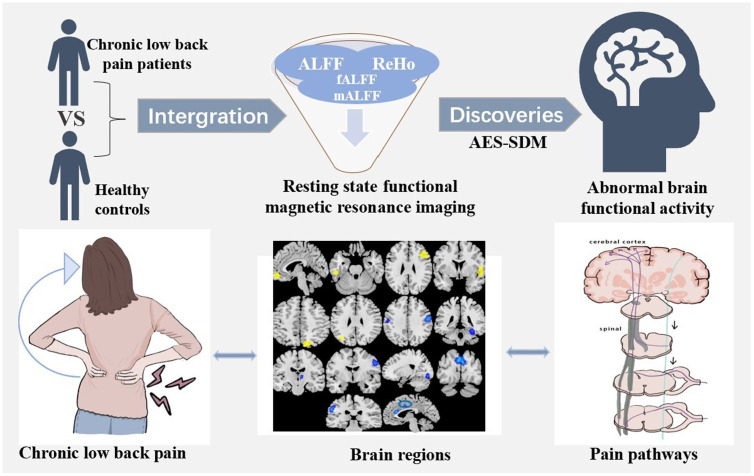

Using anisotropic effect-size signed differential mapping (AES-SDM) software, studies compared chronic low back pain (CLBP) patients with healthy controls (HCs) of resting state functional magnetic resonance imaging are integrated for the first time. Spontaneous brain activity abnormalities related to pain were found.

## 1 Introduction

Chronic low back pain (CLBP) is a pervasive musculoskeletal affliction with profound societal repercussions, ranking among the most prevalent chronic pain conditions (George et al., 2021; JOSPT, 2021). It refers to bone and muscle pain that extends from the 12th rib to the buttock fold for more than 3 months, which sometimes extends to the thighs (above the knee) (Hartvigsen et al., 2018; Shi et al., 2021). Anatomically, CLBP can arise from any innervated structures at the lumbar spine. Pain is the predominant motivator for individuals with CLBP to seek medical intervention (Mosley et al., 2017). CLBP is often closely intertwined with local pain and functional impairments, as well as with emotional and cognitive behavioral disorders, including anxiety, depression, and sleep disturbances. Globally, CLBP stands as a principal contributor to disability, with its incidence rates exhibiting an unrelenting upward trend (Global Burden of Disease Study 2013 Collaborators, 2015; Meucci et al., 2015). It also imposes a significant economic burden and leads to a substantial deterioration in the patients’ quality of life (GBD 2019 Diseases and Injuries Collaborators, 2020; Lin et al., 2022).

Recent research has yet to clarify the underlying mechanisms of CLBP, significantly impeding our comprehension of its neuropathological aspects and the development of effective therapeutic strategies. Many previous systematic reviews and clinical studies have indicated a weak or inconsistent correlation between CLBP and degenerative spinal changes, suggesting that the condition’s etiology may be more complex than initially presumed (Chou and Shekelle, 2010; Vanderstraeten et al., 2023), while increasing evidence shows that CLBP is significantly related to changes in brain function and structure (Zhang et al., 2019). Previous fMRI studies have found that patients with CLBP exhibited abnormal gray matter volume in the prefrontal cortex, the precentral gyrus and other extensive areas (Yang et al., 2017). Kregel et al. (2015) have summarized changes in the white matter of the brain specific cortical and subcortical areas in CLBP patients. Moreover, previous studies have confirmed that there are many spontaneous brain activity changes in patients with CLBP compared to Healthy Controls (HCs), such as medial prefrontal cortex (Zhou et al., 2018, 2019; Zhang et al., 2017; Sun, 2018), precuneus (Zhou et al., 2019; Chen et al., 2011; Zou et al., 2019; Hu, 2020; Zhu et al., 2020; Zhang, 2021), cerebellum posterior lobe (Zhang et al., 2017; Sun, 2018; Hu, 2020) and parahippocampal gyrus (Sun, 2018; Zhu et al., 2020). Increased ALFF and decreased ReHo in the caudate nucleus were also detected in different researches (Gao et al., 2020; Zhao, 2021). However, some studies have reported no changes in brain regions with CLBP (Li et al., 2019), and conflicting findings have also been reported in some studies (Zhou et al., 2019; Zhang et al., 2017; Hu, 2020; Gao et al., 2020; Zhao, 2016).

Resting state functional magnetic resonance imaging (rs-fMRI) is a powerful, noninvasive technique for exploring the neurological mechanisms, and has been used in many diseases, including chronic pain disorders. It can measure to neural activity by measuring changes in oxygenation concentration (BOLD signals). Common local metrics of BOLD signals include regional homogeneity (ReHo), amplitude of low-frequency fluctuation (ALFF), and its standardized variant fractional ALFF (fALFF), mean normalized low-frequency amplitude (mALFF), and functional connectivity (FC). ReHo describes the local similarity or synchronization of adjacent voxel time series, focusing on the coherence and centrality of regional activities (Ou et al., 2018), while ALFF/fALFF/mALFF depict the consistent amplitude of each signal’s time history, focusing on measuring the intensity of brain activities (Chang et al., 2021). Although FC is also a frequently utilized analytical approach, it centers on the interactions between different brain regions and typically involves the prior selection of regions of interest to investigate the connectivity patterns within brain networks. The other four methods, primarily focus on the consistency and intensity of activities within brain regions themselves. Numerous previous studies have demonstrated that ReHo and ALFF are the most commonly employed rs-fMRI data analysis techniques for capturing spontaneous neural activity within specific brain regions. Combining these two methods in the neuroimaging analytical processes can provide more comprehensive information and better explanatory power.

Anisotropic effect-size signed differential mapping (AES-SDM) is a coordinate-based meta-analysis approach used to summarize and integrate neuroimaging research results that display statistically significant differences in functional activity between patients and control groups in peak-clusters (Radua et al., 2012, 2014). Despite potential influences from raw data and publication bias, it leverages and enhances many of the positive attributes identified in other analytical approaches. It not only allows for the reconstruction of effect size and statistical parameter maps that show the functional activity increases and decreases in different brain regions among multiple original studies, but it can also perform meta-regression, sensitivity and heterogeneity analysis, and bias tests by convolving isotropic kernels with peaks. So far, AES-SDM has been widely applied in neuroimaging meta-analyses in various diseases, such as migraine, post-traumatic stress disorder, anxiety disorders and so on (Li et al., 2022; Zhang et al., 2023a; Schrammen et al., 2022), due to its high sensitivity, low false positive rates, and consistency.

Hence, this study aims to perform a meta-analysis of previously published CLBP fMRI studies by using AES-SDM method, which could ultimately obtain consistent and reliable functional brain activity alterations. At the same time, to explore the potential correlation of clinical variables such as age, disease duration, and the pain intensity with these abnormal functional activities in brain regions of CLBP.

## 2 Methods

This meta-analysis has been registered in the International Prospective Register of Systematic Reviews (PROSPERO) (registration number: CRD42023428454).

### 2.1 Search strategy

A systematic search was conducted in seven electronic databases from the inception of the databases to December 22, 2024, including PubMed, EMBASE, Cochrane Central Register of Controlled Trials (CENTRAL), Web of Science, China National Knowledge Infrastructure (CNKI), Chongqing VIP (VIP), and Wanfang Database (WF). All the publications within this date were searched without any restrictions of country or article type. Briefly, the general search terms included the following words: (“low back pain” OR “dorsalgia” OR “back disorder*” OR “coccydynia” OR “sciatica” OR “back muscles”) AND (“magnetic resonance imaging” OR “MRI” OR “functional MRI” OR “fMRI” OR “voxel wise” OR “voxel” OR “regional homogeneity” OR “ReHo” OR “amplitude of low frequency fluctuations” OR “ALFF” OR “fractional amplitude of low frequency fluctuation” OR “fALFF” or “mean normalized low-frequency amplitude” OR “mALFF”) AND (“resting state”). The search strategy was transformed to adapt to Chinese electronic databases. Additionally, to identify any potential studies that were not found in our searches, the reference lists of relevant articles and reviews were consulted as additional searches.

### 2.2 Selection criteria

This meta-analysis adheres to the Preferred Reporting Items for Systematic Reviews and Meta-Analyses (PRISMA) 2020 guidelines and checklist (Page et al., 2021). The inclusion criteria were as follows: (1) original research published in English or Chinese journals and peer-reviewed; (2) the differences of brain functional activity were compared between adult patients with CLBP and HCs; (3) the analysis methods used in researches was ReHo or ALFF, fALFF, mALFF or included all of them; (4) the standardized three-dimensional coordinates (x, y, z) of results were reported in Talairach space or Montreal Neurological Institute (MNI), or reported null findings; (5) magnetic strength of the MRI scanner was at least 1.5 Tesla.

**Figure 1:**
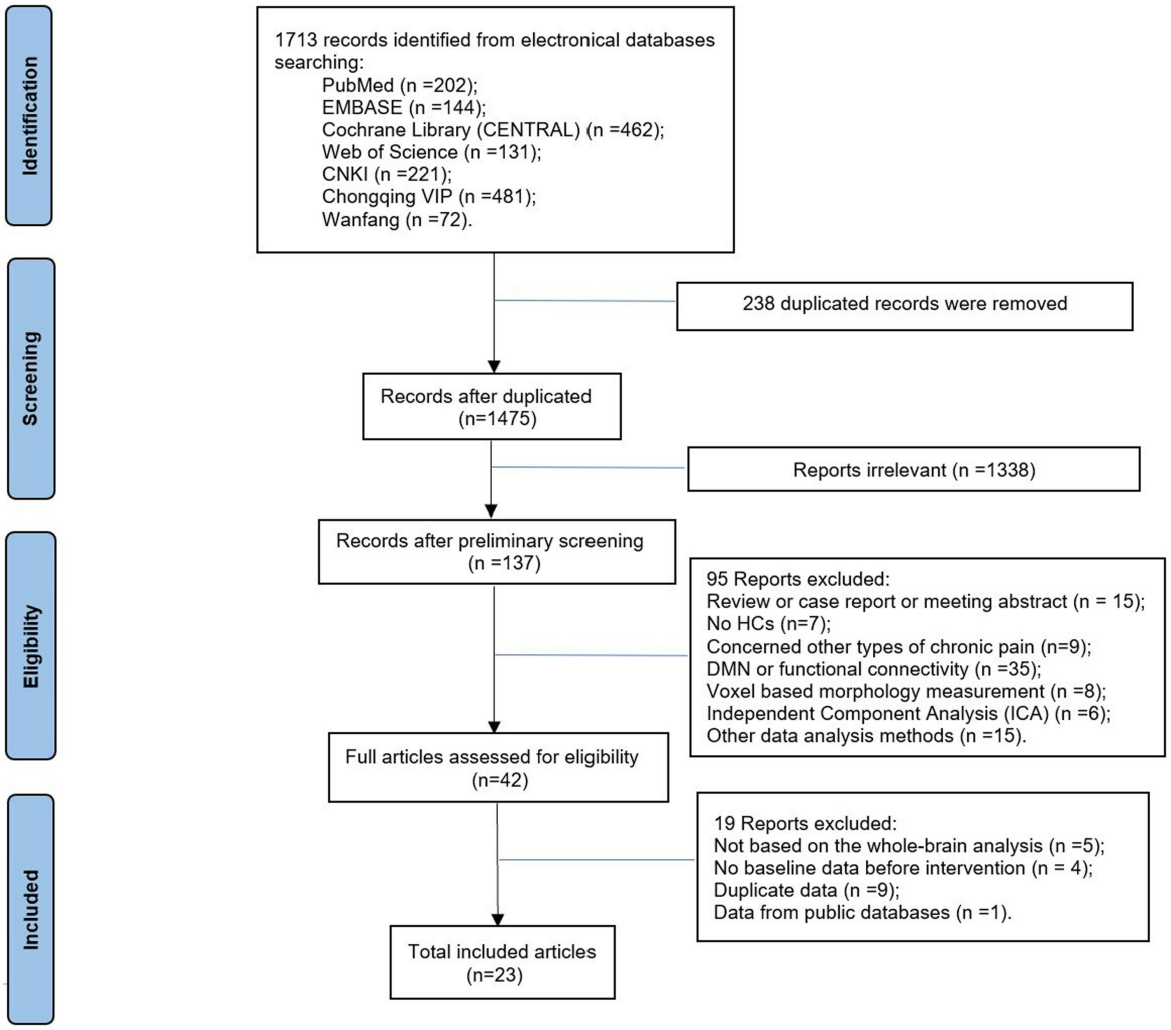
The flow diagram of the studies according to the PRISMA 2020 guidelines. n, number; PRISMA, preferred reporting items for systematic reviews and meta-analyses.

The exclusion criteria were as follows: (1) researches only reported region of interest (ROI) results; (2) article types were animal studies, case reports or conference proceedings; (3) the number of participants was less than 10 in either the patient group or control group. The detailed screening process is presented in Figure 1. Article screening was performed independently by two authors (XC, PL) and supervised by another reviewer (SC).

If two or more studies used the same data source, only the articles with the largest sample size and more comprehensive information would be included. Besides, for longitudinal or intervention studies, we only included the baseline comparison between patients and HCs.

### 2.3 Data extraction

The information of included articles was independently extracted by two authors (XC and NC). Information of each included study was extracted and presented in Table 1, including: first author, year of publication, demographics (sample size and mean age), illness duration, visual analog scale (VAS) scores, and MRI technical details (MRI scanner, FWHM, analytic methods, correction, threshold, etc.). Statistically significant coordinates in each study were independently extracted according to the requirements of AES-SDM. If coordinates were missing or statistical values were not reported, the corresponding authors of the studies would be contacted by email. If peaks or statistics were not provided from studies reporting null findings, we recorded as none. Any disagreement was discussed by the authors’ group and the result was agreed upon.

**Table 1:** Demographic, clinical characteristics and technique details of the included studies.

	Analysis	Sample size (male)	Mean age (y)	Mean duration(m)	VAS	Scanner	FWHM	Threshold	Space	Number of coordinates	Quality score
		CLBP	HC	CLBP	HC	CLBP	CLBP						
ReHo studies													
Chen et al. (2011)	ReHo	15(5)	16(6)	50.90	53.10	NA	6.47	1.5 T	4 mm	*p* < 0.05(corrected)	Talairach	7	9.0
Zhou et al. (2019)	ReHo	25(NA)	26(NA)	55.16	53.38	36.72	5.78	3 T	6 mm	*p* < 0.05(corrected)	MNI	7	9.5
Zou et al. (2019)	ReHo	32(15)	25(12)	46.37	40.01	NA	NA	3 T	8 mm	*p* < 0.05(corrected)	MNI	22	9.5
Li et al. (2019)	ReHo	23(8)	20(6)	35.00	34.00	NA	NA	3 T	4 mm	*p* < 0.05(corrected)	MNI	0	9.0
Hu (2020)	ReHo	25(13)	20(10)	51.32	52.25	46.36	6.48	3 T	4 mm	*p* < 0.05(corrected)	MNI	10	9.0
Zhu et al. (2020)	ReHo	50(26)	47(32)	50.92	48.89	84.42	6.15	3 T	6 mm	*p* < 0.05(corrected)	MNI	10	9.5
Zhao (2021)	ReHo	25(11)	24(15)	28.00	24.00	5.60	5.25	3 T	4 mm	*p* < 0.05(corrected)	MNI	5	9.5
Zhang (2021)	ReHo	25(13)	26(14)	55.16	53.38	36.72	5.78	3 T	6 mm	*p* < 0.05(corrected)	MNI	7	9.5
Xu et al. (2023)	ReHo	29(13)	31(14)	37.48	37.08	NA	4.00	3 T	6 mm	*p* < 0.05(corrected)	MNI	1	9.5
Mei et al. (2024)	ReHo	71(39)	80(42)	49.31	47.68	20.4	6.41	3 T	6 mm	*p* < 0.05(corrected)	MNI	25	10
Zuo (2024)	ReHo	33(15)	35(13)	45.97	50.85	NA	7.08	3 T	4 mm	*p* < 0.05(corrected)	MNI	2	9.5
Chen (2024)	ReHo	27(17)	28(17)	32.20	31.80	NA	5.60	3 T	6 mm	*p* < 0.05(corrected)	MNI	1	9.5
ALFF Studies													
Zhao (2016)	ALFF	12(4)	12(4)	47.90	47.20	NA	5.74	3 T	4 mm	*p* < 0.05(corrected)	Talairach	2	9.5
Zhang et al. (2017)	ALFF	13(7)	13(7)	54.23	54.00	16.00	6.23	3 T	6 mm	*p* < 0.05(uncorrected)	MNI	13	9.5
Zhou et al. (2018)	ALFF	25(13)	27(15)	55.16	52.96	37.08	5.78	3 T	6 mm	*p* < 0.05(corrected)	MNI	13	9.5
Sun (2018)	ALFF	15(8)	15(7)	51.00	51.07	16.60	6.13	3 T	6 mm	*p* < 0.05(uncorrected)	MNI	10	10
Zhang et al. (2019)	ALFF	90(38)	74(31)	34.46	32.44	83.28	NA	3 T	6 mm	*p* < 0.05(corrected)	MNI	6	9.5
Tan et al. (2019)	ALFF	20(8)	20(8)	38.95	38.45	NA	5.33	3 T	8 mm	*p* < 0.05(uncorrected)	MNI	3	9.0
Li et al. (2019)	ALFF	23(8)	20(6)	35.00	34.00	NA	NA	3 T	4 mm	*p* < 0.05(corrected)	MNI	3	9.0
Yang et al. (2017)	ALFF	62(32)	78(34)	50.20	50.90	78.00	6.50	3 T	6 mm	*p* < 0.05(corrected)	MNI	8	9.5
Tang et al. (2023)	ALFF	36(12)	36(14)	38.58	38.17	NA	6.00	3 T	6 mm	*p* < 0.05(corrected)	MNI	10	10
Xu et al. (2023)	ALFF	29(13)	31(14)	37.48	37.08	NA	4.00	3 T	4 mm	*p* < 0.05(corrected)	MNI	3	9.5
Mei et al. (2024)	ALFF	71(39)	80(42)	49.31	47.68	20.4	6.41	3 T	6 mm	*p* < 0.05(corrected)	MNI	20	10
Zuo (2024)	ALFF	33(15)	35(13)	45.97	50.85	NA	7.08	3 T	6 mm	*p* < 0.05(corrected)	MNI	2	9.5
fALFF Studies													
Wen et al. (2022)	fALFF	27(17)	28(17)	32.20	31.80	NA	5.60	3 T	6 mm	*p* < 0.05(corrected)	MNI	2	9.5
mALFF Studies													
Zhou et al. (2024a)	mALFF	19(10)	20(10)	37.47	36.05	NA	5.68	3 T	NA	*p* < 0.05(corrected)	MNI	6	8.5
Zhou et al. (2024b)	mALFF	30(14)	31(17)	37.33	35.90	NA	5.73	3 T	NA	*p* < 0.05(corrected)	MNI	5	8.5

ReHo, regional homogeneity; ALFF, amplitude of low‐frequency fluctuations; fALFF, fractional amplitude of low frequency fluctuation; mALFF, mean amplitude of low-frequency fluctuation; y, year; m, month; VAS, Visual Analog Scale; T, tesla; FWHM, full‐width at half‐maximum; NA, not available; MNI, Montreal Neurological Institute.

### 2.4 Quality assessment

The quality of the included studies was assessed based on a 10-point checklist which was used in many previous neuroimaging meta-analyses (Supplementary Tables S1A, S1B) (Guo et al., 2024; Yan et al., 2023). The checklist focused on sample size, demographic and clinical characteristics of the study populations, imaging specific methodology and the quality of the reported results. According to the completeness of published studies, this checklist was scored as full, partial or unmet, with each item receiving a score of 1, 0.5 or 0, respectively. The total score was 10 points. The assessment of each study was carried out independently by two researchers (XC and NC) in a standardized manner. For any differences, XC and NC gave their input and decided with consensus from the third researcher (ZL).

### 2.5 AES-SDM meta-analysis

AES-SDM software^1^ was used to analyze regional changes in spontaneous brain activity between patients with CLBP and HCs (Radua et al., 2012). Z-values appeared in studies were converted into T-statistics using AES-SDM official online converter.^2^ Above all, the effect-size and variance maps were recreated by combining the extracted peak coordinate information with a Gaussian sliding kernel (full-width at half-maximum = 20 mm). Next, the mean random effects were generated by calculating the study maps voxel-wise based on intra-study variability, sample size, and inter-study heterogeneity (Cheng et al., 2020). The statistical significance and default threshold parameter were set as follows: voxel threshold: *p* ≤ 0.005, peak height threshold: peak *Z* ≥ 1.000, cluster extent threshold ≥10 voxels (Radua et al., 2012). Finally, the meta-analysis effect-size map was analyzed by comparing it with a null distribution created by a permutation algorithm.

We initially conducted two separate meta-analyses for the ALFF and ReHo studies using the AES-SDM software. Subsequently, we integrated the functional group data (ALFF + ReHo) to perform another meta-analysis. For all three meta-analyses, we employed a meta-regression analysis weighted by sample size and within-study and between-study variances to assess the correlation between brain changes and clinical variables (age, disease duration, VAS scores). A leave-one-out jackknife sensitivity analysis was used to test the repeatability and robustness of these three results. The approach involves conducting multiple iterations of the same meta-analysis, each time excluding one different dataset. Ultimately, we examine the frequency with which a particular result is consistently retained across these repeated iterations. If a brain region remains significant in all or most of (≥ 50%) the combinations of studies, it can be regarded as highly replicable (Radua et al., 2012; Long et al., 2022). The same statistical significance and default threshold parameter was set as in the main analysis. Egger tests were used to detect the asymmetry of funnel plots for publication bias, where *p* < 0.05 suggested significant publication bias. Concurrently, akin to the simple overlay of meta-analytical maps in individual meta-analyses, we utilized the multimodal meta-analysis overlap feature within the software to examine the co-localization of functional abnormalities from the first two meta-analyses, taking into account the error in *p*-values, in order to assess the convergence of results across different modalities (Radua et al., 2012, 2013). This multimodal meta-analysis provides a more statistically robust overlap.

## 3 Results

### 3.1 Characteristics of included studies

As shown in Figure 1, a total of 1,713 articles were searched. Twenty-three potential eligible articles were screened in this study (Zhang et al., 2017, 2019; Zhou et al., 2018, 2019, 2024a,b; Sun, 2018; Chen et al., 2011; Zou et al., 2019; Hu, 2020; Zhu et al., 2020; Zhang, 2021; Gao et al., 2020; Zhao, 2021; Li et al., 2019; Zhao, 2016; Tan et al., 2019; Wen et al., 2022; Xu et al., 2023; Mei et al., 2024; Zuo, 2024; Chen, 2024; Tang et al., 2023). Due to only one literature on fALFF and two literatures on mALFF being retrieved, we did not conduct separate fALFF/ mALFF meta-analysis of CLBP studies. In addition, 5 articles conducted both ReHo and ALFF analysis (Zhang, 2021; Li et al., 2019; Xu et al., 2023; Mei et al., 2024; Zuo, 2024), but in one of the studies, multi band was extracted for the ALFF analysis (Zhang, 2021). So, we considered these five articles as 9 separate studies for the meta-analysis. At last, 20 articles were included in this meta-analysis (24 studies, 12 ReHo studies, 12 ALFF studies), including 653 patients with CLBP and 653 HCs. Only one study reported no significant between-group differences in brain regions (Li et al., 2019). There was no significant statistical difference in age or gender ratio between patients and HCs. The demographic characteristics, clinical variables, and technical details of these studies were shown in Table 1.

### 3.2 Results of ReHo meta-analysis

**Figure 2:**
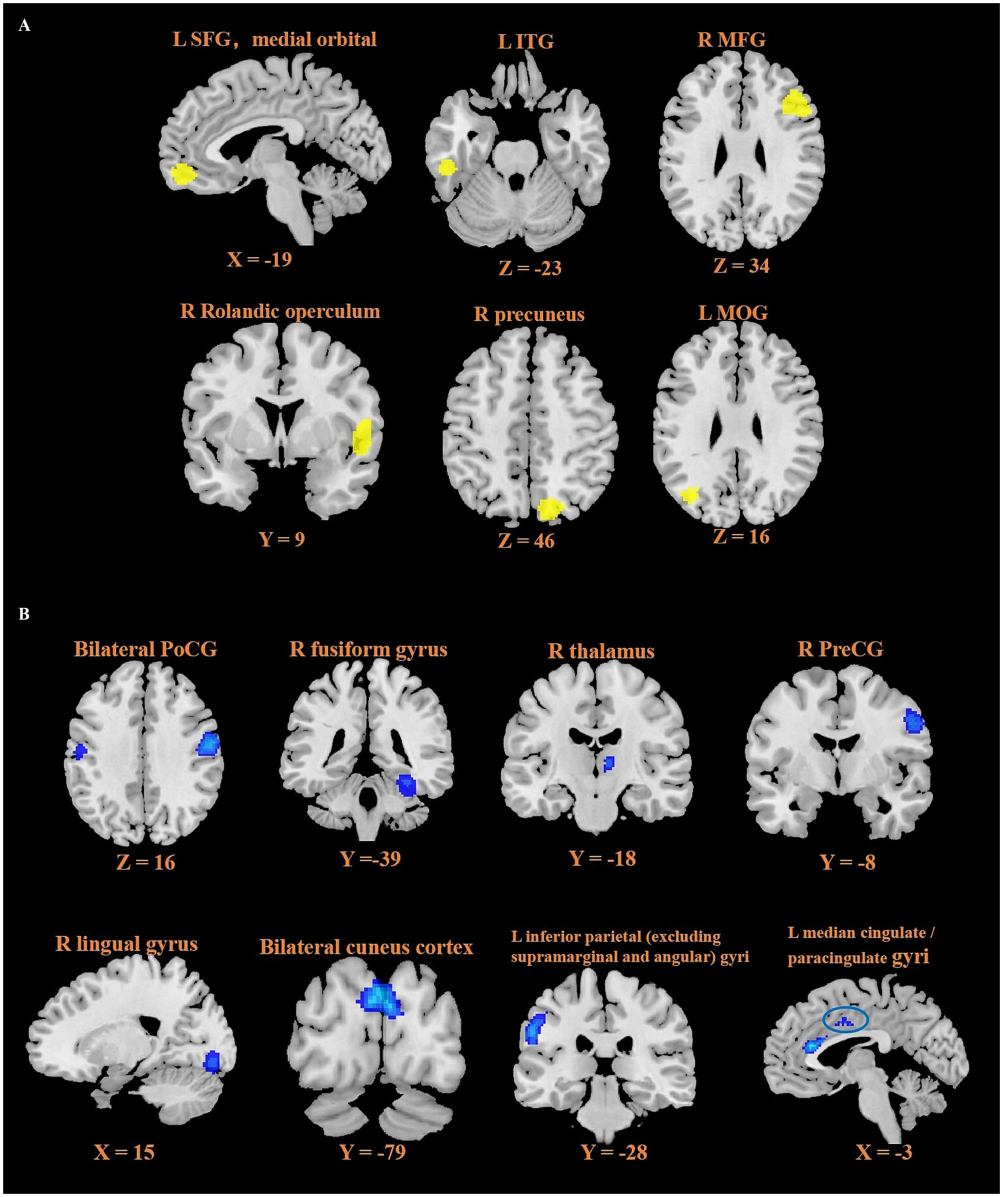
Resting-state functional difference between CLBP and HCs. Areas with increased resting-state functional activity value are displayed in yellow **(A)**. L SFG, medial orbital; L ITG; R MFG; R Rolandic operculum; R precuneus; L MOG, and areas with decreased resting-state functional activity value are displayed in blue **(B)**. Bilateral PoCG; R fusiform gyrus; R thalamus; R PreCG; R lingual gyrus; Bilateral cuneus cortex; L inferior parietal (excluding supramarginal and angular) gyri; L median cingulate / paracingulate gyri. L = left; R = right; SFG = superior frontal gyrus; ITG = inferior temporal gyrus; MFG = middle frontal gyrus; MOG = middle occipital gyrus; PoCG = postcentral gyrus; PreCG = precentral gyrus.

As illustrated in Table 2 and Figure 2, six peak foci were revealed according to the ReHo meta-analysis. Patients with CLBP had increased functional activity in the left inferior temporal gyrus (ITG, z = 2.261, *p* < 0.0001), left superior frontal gyrus (SFG), medial orbital (z = 1.962, *p* = 0.0001), right middle frontal gyrus (MFG, z = 1.603, *p* = 0.0014), right precuneus (z = 1.557, *p* = 0.0019) and decreased functional activity in the right fusiform gyrus (z = −2.162, *p* = 0.0001) and bilateral postcentral gyrus (PoCG, z = −1.991, *p* = 0.0005) compared with HCs.

**Table 2:** Meta‐analysis results of ReHo abnormalities in resting state fMRI between CLBP patients and HCs.

	MNI coordinate			SDM z-score^a^	*p* value^b^	Number of voxels^c^	Cluster breakdown (number of voxels)	Heterogeneity	Jackknife analysis
	X	Y	Z						
CLBP > HCs									
L inferior temporal gyrus	−58	−32	−22	2.261	<0.0001	552	L inferior temporal gyrus, BA20 (352)	Yes	12/12
L superior frontal gyrus, medial orbital	−6	46	−14	1.962	0.0001	546	Bilateral superior frontal gyrus, medial orbital, BA11 (270)	No	8/12
							L anterior cingulate / paracingulate gyri, BA11 (13)		
							L superior frontal gyrus, orbital part, BA11 (15)		
							R gyrus rectus, BA11 (10)		
R middle frontal gyrus	36	26	52	1.603	0.0014	50	R middle frontal gyrus, BA9 (40)	No	11/12
R precuneus	14	−72	48	1.557	0.0019	29	R precuneus, BA7 (25)	No	11/12
CLBP < HCs									
Bilateral postcentral gyrus	−42	−12	48	−1.991	0.0005	572	L precentral gyrus, BA6 (91)	No	7/12
							L postcentral gyrus, BA6, BA43, BA48, BA3, BA4 (162)		
							L supramarginal gyrus, BA48, BA43, BA3 (132)		
							R postcentral gyrus, BA43, BA3 (32)		
R fusiform gyrus	32	−34	−22	−2.162	0.0001	204	R fusiform gyrus, BA37, BA20, BA30 (123)	No	11/12
							R cerebellum, hemispheric lobule IV / V, BA20, BA37 (33)		

a Peak height threshold: z > 1.

b Voxel threshold: *p* < 0.005.

c Cluster extent threshold: regions with ≤ 10 voxels not reported in the cluster breakdown.

ReHo, regional homogeneity; CLBP, chronic low back pain; fMRI, functional magnetic resonance imaging; HCs, healthy controls; MNI, Montreal Neurological Institute; SDM, size-signed differential mapping; BA, Brodmann area; L, left; R, right.

### 3.3 Results of ALFF meta-analysis

Patients with CLBP had increased functional activity in the right rolandic operculum (extending to the right insula and right IFG) (z = 3.135, *p* < 0.0001), left ITG (z = 2.138, *p* = 0.0008), left middle occipital gyrus (MOG, z = 1.767, *p* = 0.0033), as well as decreased functional activity in the left paracentral lobule (z = −1.880, *p* = 0.0005), left postcentral gyrus (PoCG, z = −1.790, *p* = 0.0008) and bilateral cuneus cortex (z = −1.890, *p* = 0.0004) compared with HCs (Table 3; Figure 2).

**Table 3:** Meta‐analysis results of ALFF abnormalities in resting state fMRI between CLBP patients and HCs.

	MNI coordinate			SDM z-score^a^	*p* value^b^	Number of voxels^c^	Cluster breakdown (number of voxels)	Heterogeneity	Jackknife analysis
	X	Y	Z						
CLBP > HCs									
R rolandic operculum	56	4	6	3.135	<0.0001	3,854	R rolandic operculum, BA48, BA6 (382)	No	10/12
							R inferior frontal gyrus, triangular part, BA45, BA48, BA47, BA44 (545)		
							R insula, BA47, BA48, BA38 (447)		
							R superior temporal gyrus, BA48, BA22, BA21, BA38 (593)		
							R precentral gyrus, BA6 (18)		
							R inferior frontal gyrus, opercular part, BA48, BA44, BA6, BA45, BA38 (398)		
							R temporal pole, superior temporal gyrus, BA38, BA48 (162)		
							R inferior frontal gyrus, orbital part, BA47, BA38 (272)		
							R heschl gyrus, BA48 (66)		
L inferior temporal gyrus	−46	−22	−28	2.138	0.0008	442	L inferior temporal gyrus, BA 20 (243)	Yes	8/12
							L fusiform gyrus, BA 20 (22)		
L middle occipital gyrus	−34	−74	32	1.767	0.0033	17	Bilateral paracentral lobule, BA 4 (154)	No	10/12
CLBP < HCs									
L paracentral lobule	−4	−32	54	−1.880	0.0005	404	Bilateral precuneus, BA 5 (164)	No	9/12
							Bilateral paracentral lobule, BA 4, BA 5 (82)		
							L median cingulate / paracingulate gyri (49)		
Bilateral cuneus cortex	6	−78	22	−1.890	0.0004	196	R cuneus cortex, BA 18 (61)	No	8/12
							L cuneus cortex, BA 18, BA 19 (50)		
L postcentral gyrus	−36	−38	60	−1.790	0.0008	97	L postcentral gyrus, BA 2, BA 3 (85)	No	10/12

a Peak height threshold: *z* > 1.

b Voxel threshold: *p* < 0.005.

c Cluster extent threshold: regions with ≤ 10 voxels not reported in the cluster breakdown.

ALFF, amplitude of low-frequency fluctuations; CLBP, chronic low back pain; fMRI, functional magnetic resonance imaging; HCs, healthy controls; MNI, Montreal Neurological Institute; SDM, size-signed differential mapping; BA, Brodmann area; L, left; R, right.

### 3.4 Multimodal analysis of ReHo and ALFF studies

On the basis of the above meta-analyses, we utilized the multimodal functionality of the ASE-SDM software to overlay regions with altered ReHo and ALFF, and discovered increased functional activity in the left ITG of patients with CLBP.

### 3.5 Main functional meta-analysis

The results of the functional group meta-analysis (ReHo + ALFF) (Table 4; Figure 2) revealed that compared with HCs, patients with CLBP displayed new decreased functional activity in the left inferior parietal (excluding supramarginal and angular) gyri (z = −2.250, *p* < 0.0001), left median cingulate / paracingulate gyri (z = −1.936, *p* = 0.0007), right thalamus (z = −1.833, *p* = 0.0012), right precentral gyrus (PreCG, z = −1.876, *p* = 0.0010) and right lingual gyrus (z = −1.664, *p* = 0.0031).

**Table 4:** Meta‐analysis results of functional abnormalities (ReHo + ALFF) in resting state fMRI between CLBP patients and HCs.

	MNI coordinate			SDM z-score^a^	*p* value^b^	Number of voxels^c^	Cluster breakdown (number of voxels)	Heterogeneity	Jackknife analysis
	X	Y	Z						
CLBP > HCs									
L inferior temporal gyrus	−50	−18	−26	2.726	<0.0001	777	L inferior temporal gyrus, BA20 (468)	Yes	24/24
L superior frontal gyrus, medial orbital	−4	52	−10	1.805	0.0020	84	L superior frontal gyrus, medial orbital, BA11, BA10 (68)	No	19/24
R middle frontal gyrus	34	24	52	1.985	0.0008	60	R middle frontal gyrus, BA9, BA8 (52)	No	22/24
CLBP < HCs									
L inferior parietal (excluding supramarginal and angular) gyri	−56	−22	38	−2.250	<0.0001	285	L postcentral gyrus, BA4 (87)	No	21/24
							L inferior parietal (excluding supramarginal and angular) gyri, BA3 (37)		
							L supramarginal gyrus, BA2, BA48, BA3 (101)		
L median cingulate / paracingulate gyri	0	−40	54	−1.936	0.0007	281	Bilateral median cingulate / paracingulate gyri (69)	No	21/24
							Bilateral precuneus, BA5 (102)		
							Bilateral paracentral lobule, BA5, BA4 (79)		
R thalamus	6	−8	2	−1.833	0.0012	240	R striatum (106)	No	21/24
							R caudate nucleus, BA25 (22)		
							R thalamus (18)		
R postcentral gyrus	50	−24	50	−1.956	0.0006	152	R postcentral gyrus, BA3, BA4 (135)	No	21/24
R precentral gyrus	52	0	46	−1.876	0.0010	78	R precentral gyrus, BA6 (62)	No	17/24
R lingual gyrus	12	−86	−8	−1.664	0.0031	62	R lingual gyrus, BA18 (36)	No	22/24
R cuneus cortex	6	−84	24	−1.658	0.0031	18	R cuneus cortex, BA18 (12)	No	19/24

a Peak height threshold: *z* > 1.

b Voxel threshold: *p* < 0.005.

c Cluster extent threshold: regions with ≤ 10 voxels not reported in the cluster breakdown.

ReHo, regional homogeneity; ALFF, amplitude of low-frequency fluctuations; CLBP, chronic low back pain; fMRI, functional magnetic resonance imaging; HCs, healthy controls; MNI, Montreal Neurological Institute; SDM, size-signed differential mapping; BA, Brodmann area; L, Left; R, Right.

### 3.6 Heterogeneity analysis and publication bias

All meta-analyses revealed significant heterogeneity in the right ITG. Egger tests demonstrated that there was no publication bias (*p* > 0.05) in the meta-analysis of functional data (Supplementary Figures S1A, S1B).

### 3.7 Jackknife sensitivity analysis

After reading the SDM software instructions and reviewing some meta-analyses related to neuroimaging, it became evident that a consensus regarding the threshold for sensitivity analysis to assess the robustness of the primary outcome is lacking (Long et al., 2022; Wang et al., 2020). Finally, we opted to employ a threshold of 50% in our meta-analysis (Radua et al., 2012; Zhou et al., 2017). The jackknife analysis revealed that all of the aforementioned regions exhibited stability, being reliably detected across the three meta-analyses (Supplementary Tables S2–S4).

### 3.8 Meta-regression analysis

As depicted in Tables 5–7, in meta-regression analyses, we examined the potential correlations between clinical variables (age, disease duration, and VAS scores) and these abnormal brain regions. The regression analyses based on ReHo and ALFF revealed correlations between the age of CLBP patients and specific brain regions such as the right precuneus, left PoCG, and left MOG. Consistently across all three regression analyses, a correlation was observed between the ITG and patients’ VAS scores. Furthermore, in both the standalone ALFF regression analysis and the analysis of the integrated functional dataset, correlations were identified between the disease duration of patients and several brain regions, including the PoCG, MOG, ITG, MFG, and right lingual gyrus. Since the datasets were insufficient, we did not perform meta-regression analyses on other continuous variables, such as gender, BMI, medications, and so on.

**Table 5:** Meta-regression analysis of the correlation between functional alterations and clinical variables in CLBP patients of ReHo studies.

Regions	MNI coordinate			SDM z-score^a^	*p* value^b^	Number of voxels^c^	Cluster breakdown
	X	Y	Z				
Effects of age							
R precuneus	4	−38	38	−1.466	0.0026	164	R precuneus (51)
							L precuneus (50)
							R median cingulate / paracingulate gyri, BA23 (39)
							L median cingulate / paracingulate gyri (13)
Effects of VAS							
R fusiform gyrus	32	−32	−24	−1.083	0.0030	99	R fusiform gyrus, BA20 (66)
							R cerebellum, hemispheric lobule IV / V, BA20 (15)
L inferior temporal gyrus	−48	−26	−28	−2.172	0.0004	658	L inferior temporal gyrus, BA20 (365)
							L fusiform gyrus, BA20 (22)

a Peak height threshold: z > 1.

b Voxel threshold: *p* < 0.005.

c Cluster extent threshold: regions with ≤ 10 voxels not reported in the cluster breakdown.

ReHo, regional homogeneity; VAS, Visual Analog Scale; MNI, Montreal Neurological Institute; SDM, size-signed differential mapping; BA, Brodmann area; L, Left; R, Right.

**Table 6:** Meta-regression analysis of the correlation between functional alterations and clinical variables in CLBP patients of ALFF studies.

Regions	MNI coordinate			SDM z-score^a^	*p* value^b^	Number of voxels^c^	Cluster breakdown
	X	Y	Z				
Effects of age							
L postcentral gyrus	−58	−6	14	1.607	0.0036	56	L postcentral gyrus, BA48 (29)
L middle occipital gyrus	−36	−74	34	1.719	0.0023	27	L middle occipital gyrus, BA19 (22)
Effects of duration							
L postcentral gyrus	−46	−12	40	2.316	0.0025	33	L postcentral gyrus, BA4 (20)
L middle occipital gyrus	−34	−74	70	−2.338	<0.0001	657	L angular gyrus, BA39, BA7 (249)
							L middle occipital gyrus, BA19, BA7 (229)
							L inferior parietal (excluding supramarginal and angular) gyri, BA7 (23)
L inferior temporal gyrus	−48	−34	−26	−1.423	0.0025	97	L inferior temporal gyrus, BA20 (71)
Effects of VAS							
L inferior temporal gyrus	−44	−66	−6	3.021	<0.0001	855	L inferior temporal gyrus, BA37 (167)
							L inferior occipital gyrus, BA19, BA37 (274)
							L middle temporal gyrus, BA37 (92)
							L middle occipital gyrus, BA37, BA19 (103)
							L fusiform gyrus, BA37, BA19 (72)

a Peak height threshold: z > 1.

b Voxel threshold: *p* < 0.005.

c Cluster extent threshold: regions with ≤ 10 voxels not reported in the cluster breakdown.

ALFF, amplitude of low-frequency fluctuations; VAS, Visual Analog Scale; MNI, Montreal Neurological Institute; SDM, size-signed differential mapping; BA, Brodmann area; L, Left; R, Right.

**Table 7:** Meta-regression analysis of the correlation between functional alterations and clinical variables in CLBP patients of ReHo and ALFF studies.

Regions	MNI coordinate			SDM z-score^a^	*p* value^b^	Number of voxels^c^	Cluster breakdown
	X	Y	Z				
Effects of duration							
R middle frontal gyrus	34	22	50	−2.334	<0.0001	209	R middle frontal gyrus, BA8, BA7 (182)
							R superior frontal gyrus, dorsolateral, BA8 (11)
R lingual gyrus	8	−84	−12	−1.300	0.0036	14	R lingual gyrus, BA18 (13)
Effects of VAS							
L inferior temporal gyrus	−44	−66	−8	2.047	<0.0001	526	L inferior occipital gyrus, BA19 (248)
							L inferior temporal gyrus, BA37 (106)
							L middle occipital gyrus, BA37, BA19 (70)
							L fusiform gyrus, BA19, BA37 (22)
R middle frontal gyrus	34	24	52	1.088	0.0026	40	R middle frontal gyrus, BA9, BA8 (37)

a Peak height threshold: z > 1.

b Voxel threshold: *p* < 0.005.

c Cluster extent threshold: regions with ≤ 10 voxels not reported in the cluster breakdown.

ReHo, regional homogeneity; ALFF, amplitude of low-frequency fluctuations; VAS, Visual Analog Scale; MNI, Montreal Neurological Institute; SDM, size-signed differential mapping; BA, Brodmann area; L, Left; R, Right.

## 4 Discussion

This multimodal neuroimaging meta-analysis integrated information from the whole-brain ReHo and ALFF studies in CLBP by using AES-SDM software. ReHo meta-analysis showed abnormal spontaneous activity of the left ITG, left SFG, right MFG, right precuneus, right fusiform gyrus and bilateral PoCG in patients with CLBP. Meanwhile, ALFF meta-analysis demonstrated functional alterations in the right rolandic operculum, left ITG, left MOG, left paracentral lobule, left PreCG and bilateral cuneus cortex of patients with CLBP. What’s more, meta-regression analysis revealed that the age, VAS scores, duration of disease of CLBP patients were significantly associated with widespread functional abnormalities in some brain regions, such as the right precuneus, left PoCG and left ITG. The left ITG emerged as the overlapping brain region among the ReHo and ALFF studies according to the multimodal analysis. The integrated functional analysis showed the left inferior parietal (excluding supramarginal and angular) gyri, left median cingulate / paracingulate gyri, right thalamus, right PreCG and right lingual gyrus were newly identified brain regions with decreased functional activity. This study found that patients with CLBP have abnormal brain activity in pain-related pathways, which is also associated with a wide range of cognitive functions and closely related to emotional functions. These brain functional changes may be important causes of physical and mental dysfunction as well as the recurrence of clinical symptoms in patients with CLBP.

### 4.1 Patients with CLBP experience abnormal pain sensation and may develop adaptive mechanisms

This study revealed that patients with CLBP exhibit decreased brain activity in the PoCG and thalamus when compared to HCs. Pain is perceived as an imbalance between the ascending pathway and the descending pathway (De Ridder et al., 2022). The somatosensory cortex (SCC), as expressional central of the lateral “painfulness” pathway, encodes the discriminatory/sensory components of the pain. The PoCG is part of the SCC, which primarily governs somatic perception and serves as the ultimate central hub for all sensory pathways in the parietal lobe. The thalamus is considered a crucial relay station closely associated with pain perception and the descending pain modulation pathways. The thalamus projects to the insula and periaqueductal gray matter (PAG), receiving pain signals and transmitting them to the cerebral cortex, and it releases various neurotransmitters in the descending modulation pathways that affect the transmission of pain signals in the spinal cord. Additionally, regression analysis has found a correlation between the PoCG and the duration of CLBP. When lower back pain becomes chronic, the somatic perception dominated by the SCC also undergoes changes, potentially becoming an adaptive process. A recent literature review conducted for a clinical trial targeting the affective pain circuit found that the thalamus is the preferred target for most chronic pain patients during deep brain stimulation (Keifer et al., 2014; Nüssel et al., 2022). Additionally, a study assessing the feasibility and safety of a novel neurofeedback technique based on electroencephalography (EEG ISF-NF) in retraining activity within the SCC and pregenual anterior cingulate cortex (pgACC), as well as dorsal anterior cingulate cortex (dACC), has been confirmed as a safe and effective method for treating CLBP (Adhia et al., 2023). These findings suggest that patients with CLBP exhibit sensory abnormalities in the “pain” pathways and may imply the formation of an adaptive mechanism.

### 4.2 Patients with CLBP exhibit functional changes in multiple brain regions related to pain emotion and cognition

Abnormalities in the prefrontal cortex (PFC), precuneus and rolandic operculum are other important findings in our results. The SFG and MFG, located in the PFC, belong to the descending pain modulatory system and are also the key node of the central executive network. The PFC not only participates in advanced cognitive and memory functions, but also responsible for emotional modulation and stress perception. The role of the PFC in pain regulation has been proved repeatedly in both clinical and pre-clinical work (Ong et al., 2019; Ma et al., 2023; Zhang et al., 2023b). This system has been clearly established to not only inhibit but also facilitate pain (Wilder-Smith, 2011). In the descending pain modulation pathway, the PFC sends descending fibers to the PAG. The functional activity variations of the PFC directly affect the PAG and play a crucial role in pain modulation. A certain review summarized the voxel-based morphometry results of 15 types of chronic pain and showed that changes in the cerebral cortex, particularly in the PFC, occur in chronic pain conditions (May, 2011). Notably, both repetitive transcranial magnetic stimulation (rTMS) and repetitive transcranial direct current stimulation (rTDS) therapies have identified the dorsolateral PFC as a therapeutic target for pain management in clinical practice (Lefaucheur et al., 2020; Li et al., 2024; Che et al., 2021). Furthermore, substantial evidence highlights the pivotal role of the PFC in the development and perpetuation of CLBP.

Precuneus is a part of both the default mode network (DMN) and a key regions of brain activity during resting states (Buckner and DiNicola, 2019; Zhang et al., 2016). Precuneus is involved in higher cognition. DMN is a collection of brain regions that are implicated in various ‘high-level’ cognitive processes. The chronic pain and suffering from CLBP lead to the formation of adaptations and are regulated through connections between the SCC and the DMN. A substantial body of evidence from previous articles indicates that the condition of CLBP leads to functional abnormalities in multiple brain regions within the DMN, further supporting the involvement of the DMN in the emotional processing and cognitive functions associated with chronic pain. Additionally, some studies have utilized the resting-state functional connectivity of the DMN as a predictive marker for assessing the response of CLBP patients to treatment regimens, thereby establishing quantifiable benchmarks for selecting appropriate therapeutic strategies (Tu et al., 2019). Regression analysis has identified a correlation between the precuneus and age, implying a potential decline in cognitive and memory functions as age advances. Studies have demonstrated that older adults with elevated superiority illusion scores tend to have greater gray matter volume in the right precuneus and enhanced FC compared to younger and middle-aged individuals (Shidei et al., 2025). The precuneus undoubtedly assumes a complex and pivotal role in the context of CLBP.

The operculum, defined as the multimodal cortex adjacent to the insula, has a widespread connectivity. Its complex functions include autonomic, cognitive processing, sensory and motor (Mălîia et al., 2018). These functions all participate in the medial pathway. The medial “suffering” pathway, consisting of the rostral to dorsal anterior cingulate cortex and anterior insular cortex, processes the affective motivational aspect of pain. The central pathway’s ability to handle pain in patients with CLBP changes and this “suffering” sensation is further modulation by the central executive network and salience network. This upward pathway overlaps with those two networks, ultimately leading to emotional disorders such as anxiety and depression. As a crucial hub for the integration of emotion and sensation, the insula participates in the transmission and modulation of nociceptive information through its extensive connections with other brain regions. Within the descending modulatory pathway, the insular cortex is connected to multiple brain areas, including the amygdala, anterior cingulate cortex, and PAG, which are key nodes in the transmission and modulation of pain signals. For instance, the connection between the insular cortex and the PAG is involved in the descending modulation of pain. Additionally, the insular cortex engages in the emotional and cognitive aspects of pain through its connection with the anterior cingulate cortex. Studies applying fMRI on patients in pain have found that the insular cortex is one of the most frequently activated brain regions (Apkarian et al., 2005). In short, the degree of pain and the regulation of negative emotions in patients with CLBP are closely related to functional changes in the insular cortex (Labrakakis, 2023).

### 4.3 The visual cortex and motor cortex are involved in the processing of pain in CLBP

Additionally, this meta-analysis also found that patients with CLBP have reduced functional activity in the PreCG, fusiform gyrus, precuneus, and lingual gyrus, while there is increased functional activity in the ITG and MOG. Our multimodal analysis also identified abnormalities in the ITG. The PreCG is more anatomically associated with movement and is part of the primary motor cortex (M1), serving as a higher center for human motor function. Still, some proprioceptive fibers also project to the PreCG which encodes the intensity, location, and duration of pain. A recent update on the guidelines for the therapeutic use of rTMS indicates that the application of high-frequency—rTMS to the M1 on the side of pain in patients has a definite analgesic effect (Lefaucheur et al., 2020; Olechowski et al., 2023). The fusiform gyrus is closely associated with facial, bodily, and high spatial frequency stimuli. The lingual gyrus and MOG are also located in the occipital visual cortex, and together with the ITG, they are responsible for visual memory and visual processing. These regions send sensory information through sensory afferents to pain regulation systems, such as the thalamus and amygdala. There is growing evidence that the visual cortex has been participated in the processing of pain (Bush et al., 2021; Wei et al., 2019). In all the results of the regression analysis, a significant correlation between the ITG and VAS scores was found, indicating that functional changes in the ITG play an important role in the perception of pain intensity in patients with CLBP.

This meta-analysis has certain limitations. Firstly, the sample size of the study is relatively small, which leads to insufficient available data. While Müller et al. (2018) have noted that the number of experiments required for a meta-analysis largely depends on the expected effect size, analyses based on small samples should still be approached with caution. Secondly, many differences existed between imaging processing techniques and statistical thresholds as well as the demographic characteristics of patients, publication bias, etc. Moreover, the AES-SDM analysis is based on aggregated data, specifically the coordinates from published studies, rather than original data. This approach may result in less accurate findings. All of these factors may lead to biased results. For those reason, jackknife sensitivity analysis was used to evaluate the reliability and robustness of the results. Despite conducting heterogeneity analysis and Egger’s test, with the *p*-value of Egger’s test indicating no significant risk of bias, the funnel plot is not symmetrical. When there are significant differences in methodology, patient population, intervention measures, and other aspects, the funnel plot may become asymmetric, even in the absence of publication bias. Greater caution should be exercised when interpreting the results. Thirdly, due to the limited availability of relevant treatment studies, we did not conduct a meta-analysis between abnormal brain regions and studies related to therapeutic effects. The research advancements in related fields are worth further exploring in the future. Finally, this study focused solely on ReHo, ALFF, fALFF and mALFF. More articles using other data analysis methods are not included. Also, the studies included in this meta-analysis were case–control studies rather than longitudinal studies, and they only provided functional data. Any existing structural differences might potentially result in functional brain differences. Our study cannot entirely elucidate that the symptoms of CLBP might either cause brain changes, or on the contrary start from pre-existing brain spontaneous abnormalities. Further integration and analysis of brain structural and functional data and longitudinal studies can be conducted in the future to get further exploration of neuropathological mechanisms of chronic pain, and clarify the causal relationships between brain function alterations and the occurrence of CLBP.

## 5 Conclusion

Patients with CLBP have wide multimodal functional neuroimaging abnormalities, involving brain regions related to pain perception, emotional processing, cognitive functions, as well as the visual and motor cortices. These meta-analysis findings might provide a reference for the identification of potential therapeutic targets for CLBP in the brain.

## Data Availability

Publicly available datasets were analyzed in this study. This data can be found at: the datasets that support the findings of the current study are available from the corresponding authors upon reasonable request.

## Footnotes

^1^ https://www.sdmproject.com/software

^2^ http://www.sdmproject.com/utilities/?show=Statistics

## Supplementary material

Supplementary Figures S1A and S1B Funnel plots and Egger tests of main functional studies. Abbreviations: SE, standard error; **(A)** Left inferior temporal gyrus: 0.38, t: 0.50, df: 22, p: 0.622; **(B)** Left superior frontal gyrus, medial orbital: -0.61, t: -1.04, df: 22, p: 0.310; **(C)** Right middle frontal gyrus: -1.59, t: -2.54, df: 22, p: 0.119; **(D)** Left inferior parietal (excluding supramarginal and angular) gyri: 1.32, t: 1.78, df: 22, p: 0.088; **(E)** Left median cingulate / paracingulate gyri: -0.18, t: -0.24, df: 22, p: 0.810; **(F)** Right thalamus: 0.56, t: 0.84, df: 22, p: 0.411; **(G)** Right postcentral gyrus: 0.50, t: 0.66, df: 22, p: 0.517; **(H)** Right precentral gyrus: 0.41, t: 0.54, df: 22, p: 0.597; **(I)** Right lingual gyrus: 0.23, t: 0.29, df: 22, p: 0.772; **(J)** Right cuneus cortex: 0.32, t: 0.58, df: 22, p: 0.571.
