# Supplementary figures and images for: Multimodal abnormalities of brain function in chronic low back pain: a systematic review and meta-analysis of neuroimaging studies
Source: Front Neurosci. 2025 Feb 5;19:1535288. doi: 10.3389/fnins.2025.1535288 (PMC11836031; doi:10.3389/fnins.2025.1535288)

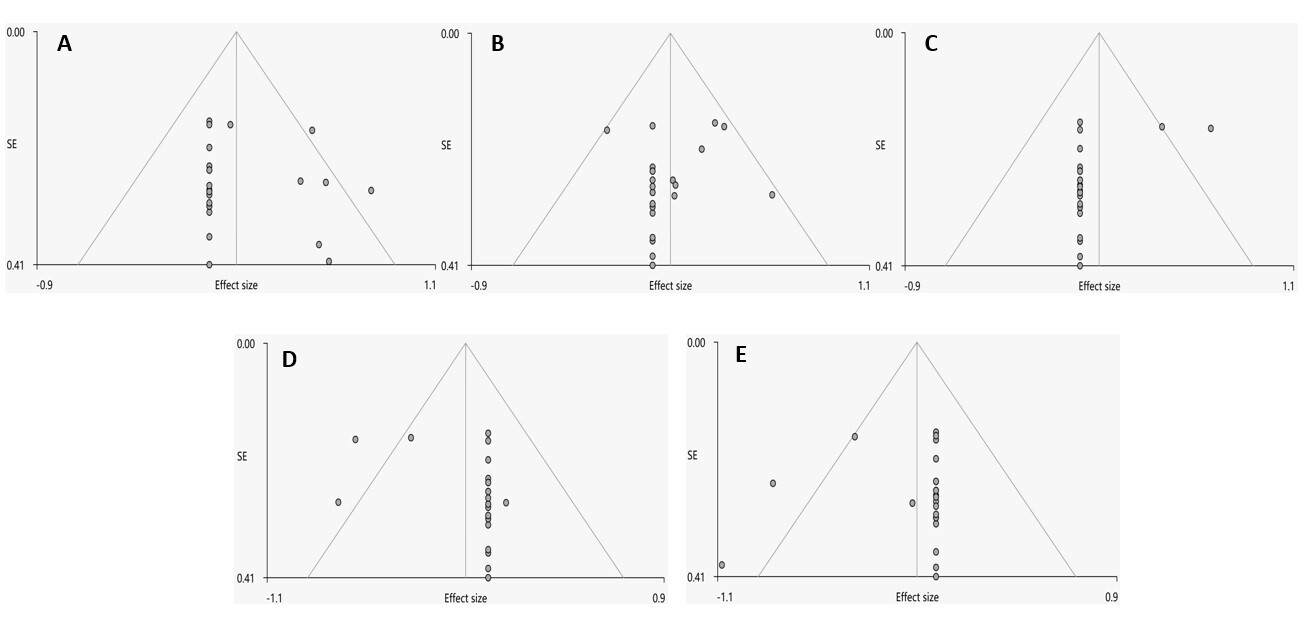

Supplement: Supplementary Figures S1A and S1B — Funnel plots and Egger tests of main functional studies. Abbreviations: SE, standard error; (A) Left inferior temporal gyrus: 0.38, t: 0.50, df: 22, p: 0.622; (B) Left superior frontal gyrus, medial orbital: -0.61, t: -1.04, df: 22, p: 0.310; (C) Right middle frontal gyrus: -1.59, t: -2.54, df: 22, p: 0.119; (D) Left inferior parietal (excluding supramarginal and angular) gyri: 1.32, t: 1.78, df: 22, p: 0.088; (E) Left median cingulate / paracingulate gyri: -0.18, t: -0.24, df: 22, p: 0.810; (F) Right thalamus: 0.56, t: 0.84, df: 22, p: 0.411; (G) Right postcentral gyrus: 0.50, t: 0.66, df: 22, p: 0.517; (H) Right precentral gyrus: 0.41, t: 0.54, df: 22, p: 0.597; (I) Right lingual gyrus: 0.23, t: 0.29, df: 22, p: 0.772; (J) Right cuneus cortex: 0.32, t: 0.58, df: 22, p: 0.571. [file Image_1.jpeg]

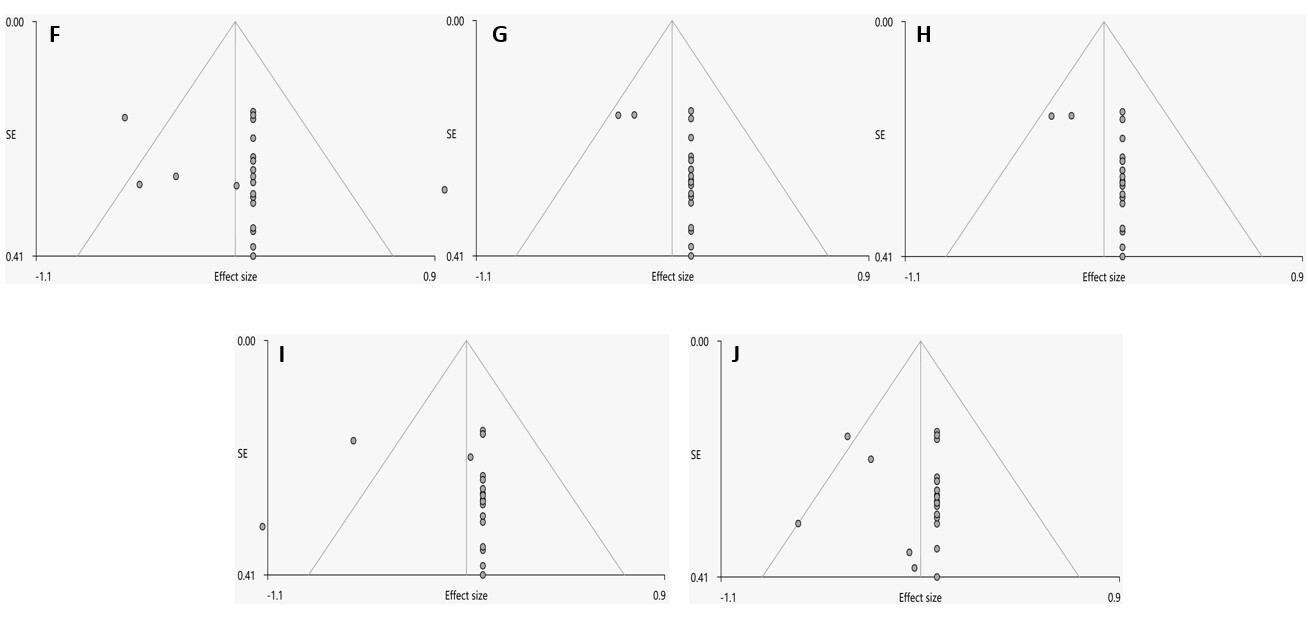

Supplement: Supplementary file 2 [file Image_2.jpeg]
