# Supplementary material for: Multimodal abnormalities of brain function in chronic low back pain: a systematic review and meta-analysis of neuroimaging studies
Source: Front Neurosci. 2025 Feb 5;19:1535288. doi: 10.3389/fnins.2025.1535288 (PMC11836031; doi:10.3389/fnins.2025.1535288)
Supplement: Supplementary file 3 [file Table_1.docx]

**Table S1A Quality Assessment Checklist (1 point per criterion fully satisfied, 0.5 for partially satisfied, 0 for otherwise).**

| **Category 1: Participants**  Score per item (0/0.5/1) |
| --- |
| 1. Patients were evaluated prospectively, specific diagnostic criteria were applied, and demographic data were reported. |
| 2. Healthy comparison participants were evaluated prospectively, and psychiatric and medical illnesses were excluded. |
| 3. Important variables (e.g., age, sex, illness duration, onset, medication status, comorbidity, severity of illness) were checked either by stratification or statistically. |
| 4. Sample size per group > 10. |
| **Category 2: Methods for image acquisition and analysis** |
| 5. Whole brain analysis was automated with no a priori regional selection. |
| 6. Coordinates reported in a standard space. |
| 7. The imaging technique used was clearly described so that it could be reproduced. |
| 8. Measurements were clearly described so that they could be reproduced. |
| **Category 3: Results and conclusions** |
| 9. Statistical parameters for significant and important nonsignificant differences were provided. |
| 10. Conclusions were consistent with the results obtained and the limitations were discussed. |
| **TOTAL** /10 points |

**Table S1B** **The quality assessment scores of included studies.**

| Study | 1 | 2 | 3 | 4 | 5 | 6 | 7 | 8 | 9 | 10 | Total |
| --- | --- | --- | --- | --- | --- | --- | --- | --- | --- | --- | --- |
| Chen et al. (2011) | 1 | 1 | 0.5 | 1 | 1 | 1 | 1 | 1 | 1 | 0.5 | 9.0 |
| Zhou et al. (2019) | 1 | 0.5 | 1 | 1 | 1 | 1 | 1 | 1 | 1 | 1 | 9.5 |
| Zou et al. (2019) | 1 | 1 | 0.5 | 1 | 1 | 1 | 1 | 1 | 1 | 1 | 9.5 |
| Li et al. (2019) | 1 | 1 | 0.5 | 1 | 1 | 1 | 1 | 1 | 0.5 | 1 | 9.0 |
| Hu et al. (2020) | 1 | 0.5 | 1 | 1 | 1 | 1 | 1 | 1 | 1 | 0.5 | 9.0 |
| Zhu et al. (2020) | 1 | 0.5 | 1 | 1 | 1 | 1 | 1 | 1 | 1 | 1 | 9.5 |
| Zhao et al. (2021) | 1 | 1 | 1 | 1 | 1 | 1 | 1 | 1 | 1 | 0.5 | 9.5 |
| Zhang et al. (2021) | 1 | 0.5 | 1 | 1 | 1 | 1 | 1 | 1 | 1 | 1 | 9.5 |
| Xu et al. (2023) | 1 | 1 | 0.5 | 1 | 1 | 1 | 1 | 1 | 1 | 1 | 9.5 |
| Mei et al. (2024) | 1 | 1 | 1 | 1 | 1 | 1 | 1 | 1 | 1 | 1 | 10 |
| Zuo et al. (2024) | 1 | 1 | 0.5 | 1 | 1 | 1 | 1 | 1 | 1 | 1 | 9.5 |
| Chen et al. (2024) | 1 | 1 | 0.5 | 1 | 1 | 1 | 1 | 1 | 1 | 1 | 9.5 |
| Zhao et al. (2016) | 1 | 1 | 0.5 | 1 | 1 | 1 | 1 | 1 | 1 | 1 | 9.5 |
| Zhang et al. (2017) | 1 | 1 | 1 | 1 | 1 | 1 | 0.5 | 1 | 1 | 1 | 9.5 |
| Zhou et al. (2018) | 1 | 0.5 | 1 | 1 | 1 | 1 | 1 | 1 | 1 | 1 | 9.5 |
| Sun et al. (2018) | 1 | 1 | 1 | 1 | 1 | 1 | 1 | 1 | 1 | 1 | 10 |
| Zhang et al. (2019) | 1 | 0.5 | 1 | 1 | 1 | 1 | 1 | 1 | 1 | 1 | 9.5 |
| Tan et al. (2019) | 1 | 1 | 1 | 1 | 1 | 1 | 0.5 | 1 | 1 | 0.5 | 9.0 |
| Gao et al. (2020) | 1 | 1 | 0.5 | 1 | 1 | 1 | 1 | 1 | 1 | 1 | 9.5 |
| Tang et al. (2023) | 1 | 1 | 1 | 1 | 1 | 1 | 1 | 1 | 1 | 1 | 10 |
| Wen et al. (2022) | 1 | 1 | 0.5 | 1 | 1 | 1 | 1 | 1 | 1 | 1 | 9.5 |
| Zhou et al. (2024) | 1 | 0.5 | 0.5 | 1 | 1 | 1 | 0.5 | 1 | 1 | 1 | 8.5 |
| Zhou et al. (2024) | 1 | 0.5 | 0.5 | 1 | 1 | 1 | 0.5 | 1 | 1 | 1 | 8.5 |

**Table S2 Sensitivity analysis of ReHo meta-analysis.**

|  | **Increased Regions** | | | | | **Decreased Regions** | |
| --- | --- | --- | --- | --- | --- | --- | --- |
|  | L inferior temporal gyrus | L superior frontal gyrus, medial orbital | | R middle frontal gyrus | R precuneus | Bilateral postcentral gyrus | R fusiform gyrus |
| Chen et al. (2011) | YES | YES | YES | | YES | YES | YES |
| Zhou et al. (2019) | YES | YES | YES | | YES | YES | YES |
| Zou et al. (2019) | YES | NO | YES | | YES | NO | YES |
| Li et al. (2019) | YES | NO | YES | | YES | YES | YES |
| Hu et al. (2020) | YES | YES | YES | | YES | YES | YES |
| Zhu et al. (2020) | YES | YES | YES | | YES | Only L | YES |
| Zhao et al. (2021) | YES | NO | YES | | YES | YES | YES |
| Zhang et al. 2021) | YES | YES | YES | | YES | YES | YES |
| Xu et al. (2023) | YES | YES | YES | | YES | Only L | YES |
| Mei et al. (2024) | YES | NO | NO | | NO | NO | NO |
| Zuo et al. (2024) | YES | YES | YES | | YES | Only L | YES |
| Chen et al. (2024) | YES | YES | YES | | YES | YES | YES |
|  | 12/12 | 8/12 | 11/12 | | 11/12 | 7/12 | 11/12 |

Abbreviations: L, left; R, right; ReHo, regional homogeneity.

**Table S3 Sensitivity analysis of ALFF meta-analysis.**

|  | Increased Regions | | | Decreased Regions | | |
| --- | --- | --- | --- | --- | --- | --- |
|  | R rolandic  operculum | L inferior temporal  gyrus | L middle occipital gyrus | L paracentral lobule | Bilateral cuneus cortex | L postcentral gyrus |
| Zhao et al. (2016) | YES | YES | YES | YES | YES | YES |
| Zhang et al. (2017) | YES | NO | YES | YES | YES | YES |
| Zhou et al. (2018) | YES | YES | NO | YES | YES | NO |
| Sun et al. (2018) | YES | NO | YES | YES | YES | YES |
| Zhang et al. (2019) | NO | NO | YES | YES | YES | YES |
| Tan et al. (2019) | YES | YES | YES | NO | YES | YES |
| Li et al. (2019) | YES | YES | YES | YES | NO | YES |
| Gao et al. (2020) | NO | YES | YES | YES | Only L | YES |
| Tang et al. (2023) | YES | YES | YES | NO | Only L | YES |
| Xu et al. (2023) | YES | YES | YES | YES | YES | YES |
| Mei et al. (2024) | YES | NO | NO | NO | NO | NO |
| Zuo et al. (2024) | YES | YES | YES | YES | YES | YES |
|  | 10/12 | 8/12 | 10/12 | 9/12 | 8/12 | 10/12 |

Abbreviations: L, left; R, right; ALFF, amplitude of low‐frequency fluctuations.

**Table S4 Sensitivity analysis of resting-state functional (ReHo + ALFF) meta-analysis.**

|  | Increased Regions | | | Decreased Regions | | | | | | |
| --- | --- | --- | --- | --- | --- | --- | --- | --- | --- | --- |
|  | L inferior temporal gyrus | L superior frontal gyrus, medial orbital | R middle frontal gyrus | L inferior parietal (excluding supramarginal and angular) gyri | L median cingulate / paracingulate gyri | R thalamus | R postcentral gyrus | R precentral gyrus | R lingual gyrus | R cuneus cortex |
| Chen et al. (2011) | YES | YES | YES | YES | NO | YES | YES | YES | YES | YES |
| Zhou et al. (2019) | YES | YES | YES | YES | YES | YES | YES | NO | YES | NO |
| Zou et al. (2019) | YES | YES | YES | NO | YES | NO | NO | YES | YES | YES |
| Li et al. (2019) | YES | YES | YES | YES | YES | YES | YES | YES | YES | YES |
| Hu et al. (2020) | YES | NO | YES | YES | YES | YES | YES | YES | NO | YES |
| Zhu et al. (2020) | YES | NO | YES | YES | YES | YES | YES | YES | YES | NO |
| Zhao et al. (2021) | YES | YES | YES | YES | YES | YES | YES | YES | YES | YES |
| Zhang et al. (2021) | YES | YES | YES | YES | YES | YES | YES | NO | YES | YES |
| Xu et al. (2023) | YES | YES | YES | YES | YES | YES | YES | YES | YES | YES |
| Mei et al. (2024) | YES | NO | NO | NO | YES | NO | YES | NO | YES | YES |
| Zuo et al. (2024) | YES | YES | YES | YES | YES | YES | YES | YES | YES | YES |
| Chen et al. (2024) | YES | YES | YES | YES | YES | YES | YES | YES | YES | YES |
| Zhao et al. (2016) | YES | YES | YES | YES | YES | YES | YES | YES | YES | YES |
| Zhang et al. (2017) | YES | YES | YES | YES | YES | YES | YES | NO | YES | YES |
| Zhou et al. (2018) | YES | NO | YES | NO | YES | NO | YES | YES | YES | YES |
| Sun et al. (2018) | YES | YES | YES | YES | YES | YES | YES | YES | YES | NO |
| Zhang et al. (2019) | YES | NO | YES | YES | YES | YES | NO | YES | YES | YES |
| Tan et al. (2019) | YES | YES | YES | YES | YES | YES | YES | YES | YES | YES |
| Li et al. (2019) | YES | YES | YES | YES | YES | YES | YES | YES | YES | NO |
| Gao et al. (2020) | YES | YES | YES | YES | YES | YES | YES | NO | NO | YES |
| Tang et al. (2023) | YES | YES | YES | YES | NO | YES | YES | NO | YES | YES |
| Xu et al. (2023) | YES | YES | YES | YES | YES | YES | YES | YES | YES | YES |
| Mei et al. (2024) | YES | YES | NO | YES | NO | YES | NO | NO | YES | NO |
| Zuo et al. (2024) | YES | YES | YES | YES | YES | YES | YES | YES | YES | YES |
|  | 24/24 | 19/24 | 22/24 | 21/24 | 21/24 | 21/24 | 21/24 | 17/24 | 22/24 | 19/24 |

Abbreviations: L, left; R, right; ReHo, regional homogeneity; ALFF, amplitude of low‐frequency fluctuations.
